# Mechanism of action of the SWI/SNF family complexes

**DOI:** 10.1080/19491034.2023.2165604

**Published:** 2023-01-12

**Authors:** Kangjing Chen, Junjie Yuan, Youyang Sia, Zhucheng Chen

**Affiliations:** a MOE Key Laboratory of Protein Science, Tsinghua University, Beijing, P.R. China; b School of Life Sciences, Tsinghua University, Beijing, P.R. China; c Tsinghua-Peking Joint Center for Life Sciences, Beijing Advanced Innovation Center for Structural Biology, Beijing, Beijing, China

**Keywords:** Chromatin remodeling, gene expression, SWI/SNF, RSC, BAF, PBAF, nucleosome recognition, transcription factor, cancer, neurodevelopmental disorder

DNA wraps around histone octamers to assemble nucleosomes, which pack into high-ordered chromatin structure in the nucleus, and block access to the genetic material. The SWI/SNF family chromatin remodeling complexes are multi-subunit, ATP-dependent molecular machines that slide and evict nucleosomes. They play a major role in control of the chromatin structure and regulate gene transcription in eukaryotic cells. Mutations of the human complexes are often found in cancers and neurodevelopmental disorders. Here, we review recent studies of the structures of the yeast complexes (SWI/SNF and RSC) and the human homologs (BAF and PBAF). We discuss how the subunits interweave into functional modules and submodules. We discuss the conserved mode of nucleosome recognition across evolution, and propose the mechanism of chromatin targeting through interaction with transcription factors and histone modifications. We also discuss the implications of disease-associated mutations in the regulation of chromatin remodeling. These findings shed light on the mechanism and function of the SWI/SNF family complexes, and provide the structural basis for drug development.

## Introduction

About 147 base-pair DNA wraps around a histone octamer to form the nucleosome, the fundamental repeating unit of chromatin [1]. Various nuclear transactions, such as gene expression, DNA replication, homologous recombination, and DNA damage repair, have to overcome the nucleosome blockage in order to get access to the DNA. Chromatin remodelers couple the energy of ATP hydrolysis to alter the position of nucleosomes and edit the histone components, playing fundamental roles in the regulation of chromatin structure and various nuclear processes [2].

Yeast Swi2/Snf2 is the first chromatin remodeling motor protein discovered almost four decades ago [3,4]. Although the motor remodels nucleosomes in vitro on its own, it functions in complexes with multiple auxiliary subunits in vivo to provide regulation and targeting. Snf2 interacts with 11 auxiliary subunits to form the SWI/SNF complex, which regulates the expression of ~5% of the genes in yeast [5]. RSC is a closely related homolog of SWI/SNF, and slide the +1 nucleosome away from the transcription start site, controlling a majority of promoter chromatin architecture in yeast [6]. The yeast SWI/SNF and RSC complexes are highly conserved across evolution, and related to the mammalian SWI/SNF (mSWI/SNF), the BAF and PBAF complexes, respectively [7]. Recently, a non-canonical SWI/SNF complex, GBAF (also named as ncBAF), is identified in human cells [8,9]. Importantly, mutations of the genes encoding the subunits of the human SWI/SNF complexes frequently occur in cancer, neurodevelopment disorder, and other diseases, in line with their broad roles in gene regulation and cell fate determination [10–12]. There is increasing interest in the therapeutic implications of the mutations of the BAF and PBAF subunits.

At the heart of the remodeling complexes lie the motor subunits, Snf2 in SWI/SNF, Sth1 in RSC, and Brg1 (also named as SMARCA4) and BRM (SMARCA2) in mSWI/SNF. The complexes contain actin and actin-related proteins (ARPs), which function in the regulation of the motor activity [13]. The complexes also contain many auxiliary subunits carrying chromatin targeting elements. For instance, Snf6 in SWI/SNF contains a SWIB domain, and is responsible for binding to transcription factors [14]. The Rsc3 and Rsc30 subunits in RSC are transcription factors themselves, and contain Zn-cluster domains (ZnD) for DNA binding [15,16]. The PBRM1, PHF10, and BRD7 subunits in PBAF contain multiple histone-tail-binding domains. How multiple subunits are assembled into the megadalton complexes, and function in nucleosome recognition and chromatin remodeling is a long-standing, fascinating question.

The structures of the canonical SWI/SNF family complexes, including yeast SWI/SNF [17–19] and RSC [20–22], and human BAF [23,24] and PBAF [25,26], were determined in recent years. Here, we discuss the emerging common theme and distinct features in the complex assembly, nucleosome recognition, and chromatin targeting of these enzymes.

## Overall modularity

Because of the complex nature and technical difficulty, the structures of the SWI/SNF family complexes remain elusive for a long time [27–33]. Ye et al. reported the first high-resolution structure of the RSC complex bound to the nucleosome [20]. This work was companied by a wave of structural determination of the related complexes, including the yeast SWI/SNF, human BAF, and most recently the human PBAF complex, in the presence and absence of the nucleosome.

**Figure 1.**
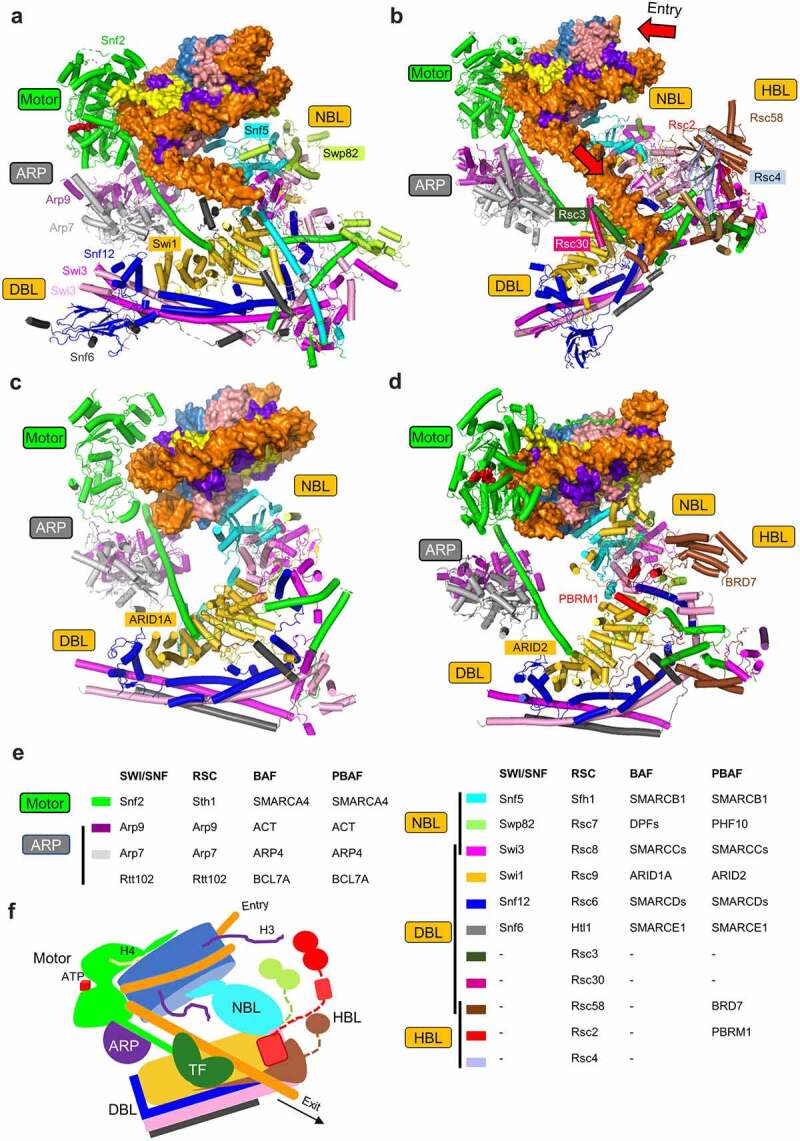
**Structures and compositions of the yeast SWI/SNF, RSC, and human BAF and PBAF complexes**. (a-d) Overall structure and modularity of the SWI/SNF (a), RSC (b), BAF (c), and PBAF (d) complexes bound to the nucleosome. The structures are derived from PBD codes 7C4J and 7EGP for SWI/SNF; 6V8O, 6TDA and 6KW4 for RSC; 6LTJ for BAF; 7VDV for PBAF. The homologous subunits are color-coded. The bound nucleotides in SWI/SNF and PBAF are shown as spheres. The direction of DNA translocation is illustrated by the arrows. For clarity, only the featured subunits of RSC, BAF and PBAF are labeled. (e) Subunit components of the canonical SWI/SNF family complexes. Swi3 in SWI/SNF, Rsc8 in RSC and SMARCC1/2 in the BAF and PBAF complexes form a dimer. Taf14 and Snf11 are presence in SWI/SNF, but their position within the complex is unclear. (f) A general model of the assembly and the mechanism of action of the SWI/SNF family complexes.

Given the sequence and function conservation, it is not surprising that the SWI/SNF family complexes share a general mechanism of action, and are organized into a similar overall architecture (Figure 1a-d). The complexes are consisted of 9–16 subunits, many of which are highly conserved from yeast to humans (Figure 1e). Based on the functionality, the complexes are divided into three modules: the motor module, the regulatory ARP module, and the substrate recruitment module (SRM). A majority of the auxiliary subunits are interweaved into the SRM, which is delineated into lobe-like submodules for nucleosome recruitment, including the nucleosome binding lobe (NBL), the DNA binding lobe (DBL), and the histone-tail binding lobe (HBL). The NBL and DBL are shared by all of the SWI/SNF family complexes, whereas the HBL is unique to RSC and PBAF. Whereas the motor and ARP modules show marked resemblance across different SWI/SNF complexes, the SRM confers the complexes with the specific features in chromatin targeting and transcriptional responses.

The motor-regulation-recruitment tripartite modularity provides the framework to understand the individual and collective functions of the components of the SWI/SNF family complexes (figure 1f). Whereas the motor module is responsible for the ultimate DNA translocation reaction, its activity is regulated by the ARP module. Many of auxiliary subunits are interwoven into the recruitment module, which functions to integrate various chromatin clues, including transcription factors and histone modifications, to target the motor in control of chromatin structure and gene transcription. This model of the complex also provides a basis to understand the different mechanisms of deregulation under disease conditions.

## Nucleosome engagement by the motor

**Figure 2.**
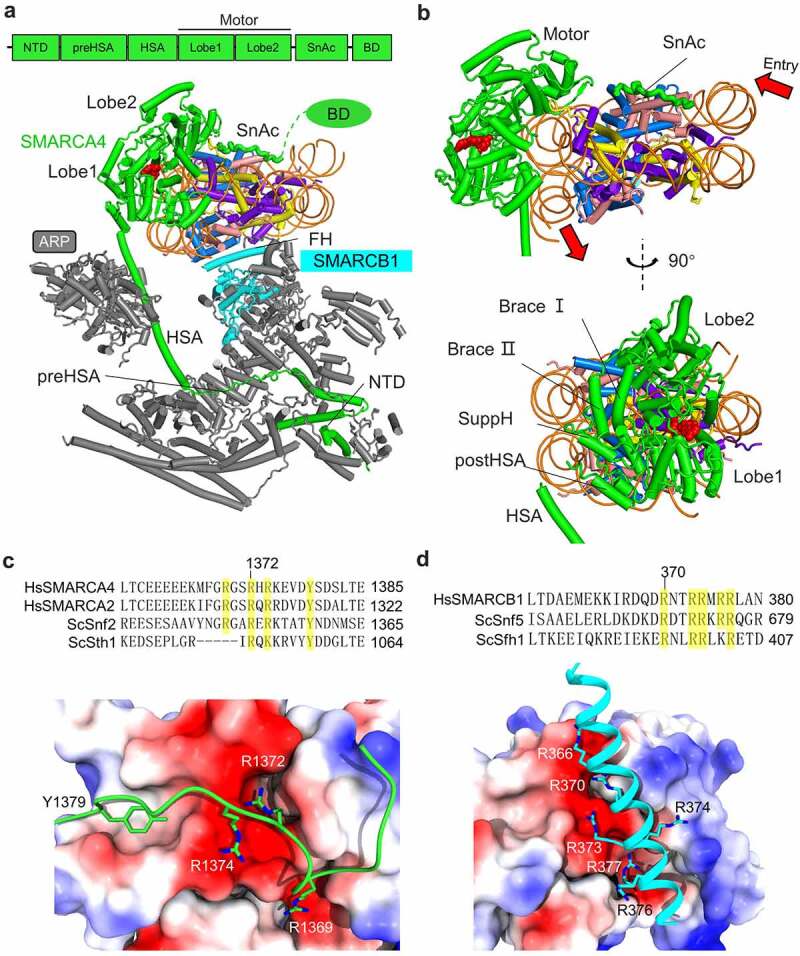
**Mechanism of nucleosome recognition**. (a) Nucleosome binding of the PBAF complex. Top panel, domain organization of the motor subunit. (b) Two views of nucleosome engagement by the motor. (c) Mechanism of nucleosome recognition by the SnAc domain of SMARCA4. Top panel, multi-sequence alignments around the arginine anchor region of the SnAc domain. (d) Mechanism of nucleosome recognition by the finger helix of SMARCB1. Top panel, multi-sequence alignments around the finger helix region.

The motor module is composed of the ATPase motor domains of the catalytic subunits, which are highly conserved and drive the nucleosome sliding reaction. The motor interacts with the nucleosome through the DNA, the H2A-H2B acidic patch and the H4 tail (Figure 2a). The motor-DNA interaction in an ATPase cycle powers the fundamental reaction of chromatin remodeling, the mechanism of which has been extensively studied and reviewed elsewhere [34,35]. The nucleosome is pseudo-symmetrical, and the binding position of the motor dictates the direction of DNA translocation (Figure 2b). The motor pulls the DNA in from the proximal end of the DNA gyre it binds, and pushes the DNA out to the distal end [36]. In the reported structures of the BAF complex, the motor domains loosely bind the nucleosome, and are in the inactive states [23,24]. This calls for prudence to extract biological implication from the structure by considering both the local resolution and the fundamental biochemistry.

The motor subunits are multiple domain proteins, and work as a global organizer to coordinate the actions of all the auxiliary subunits into one holo-enzyme (Figure 2a). Lobe1 and Lobe2 form the central catalytic motor, and are flanked by the N-terminal domain (NTD), preHSA and HSA domains at the N-terminus, and by the SnAc domain and bromodomain (BD) at the C-terminus. The NTD and the preHSA domain interact with the targeting subunits to form the SRM; the HSA helix binds the ARP proteins; the SnAc domain interacts with the nucleosome, promoting the nucleosome sliding reaction; the C-terminal BD is tethered to the complex through a flexible linker and disordered in the structures.

The polybasic SnAc domain is highly conserved, extends from the motor domains, binds to the discface of the nucleosome (Figure 2c), and functions to couple ATP hydrolysis to nucleosome sliding [37,38]. The mechanism of SnAc in nucleosome recognition is illustrated by the recent high-resolution structure of the PBAF-nucleosome complex [25]. Three arginine residues of the SnAc domain recognize the acidic patch of H2A-H2B, which is a common hub for binding of varied chromatin factors [39]. Arg1372 in SMARCA4, equivalent to Arg1352 in Snf2 and Arg1051 in Sth1, serves as the canonical arginine anchor to insert into the deep acidic pocket of H2A-H2B. Weak EM densities of the SnAc domains were identified in the previous structures of SWI/SNF [18], RSC [21], miniRSC [40], and human BAF [23], supporting a general mechanism of nucleosome recognition by the Snf2-family motors.

## Regulation of the motor by the ARP module

The motor activity is regulated by the ARP module, which is mainly composed of actin and ARPs, Arp7 and Arp9 in the yeast complexes, and ACTIN and ARP4 in the human complexes (Figure 1e). The binding of Arp7 and Arp9 is stabilized by the small subunit Rtt102 [41]. It seems that BCL7A in the human complexes plays a similar role [26]. The ARP module is positioned adjacent to the motor (Figure 2a), and functions not only to connect the SRM and the motor but play an important role in regulation of the motor activity. Loss of Arp7 or Arp9 is lethal in yeast [42]. The lethal phenotypes are suppressed by the mutations clustering at the HSA, postHSA, and SuppH of the Sth1 motor, which deregulate the nucleosome remodeling activity of Sth1 in vitro [13,43]. In line with the notion of motor regulation, the activity of Snf2 motor is reduced by the HSA domain and the formation of Arp7-Arp9-Rtt102-Snf2 tetramer [44]. A recent study indicates that the ARP proteins modulate the helical conformations of HSA and the adjacent postHSA [40], which is a part of the regulatory hub [45] (Figure 2b). Together, these studies suggest that the ARP module controls the remodeling activity through tuning the structure of the regulatory hub of the motor.

The regulatory hub is formed by the postHSA, SuppH, Brace I, and Brace II helices, which are aligned in space across the Lobe1-Lobe2 interface in the activated state of the motor (Figure 2b). The Brace I and Brace II helices are disordered in the resting state of the enzyme [44]. Upon binding to the nucleosome, they adopt the helical conformation, and interact with SuppH of Lobe1^36^. The Brace helices mediate the interaction between the two RecA-like lobes and are important for coupling ATP hydrolysis to DNA translocation [36]. The importance for the regulation of the motor activity underlies the high mutational frequency of the regulatory hub observed in cancer patients (more discussion below).

## Recognition of the H2A-H2B acidic patch by the NBL

**Figure 3.**
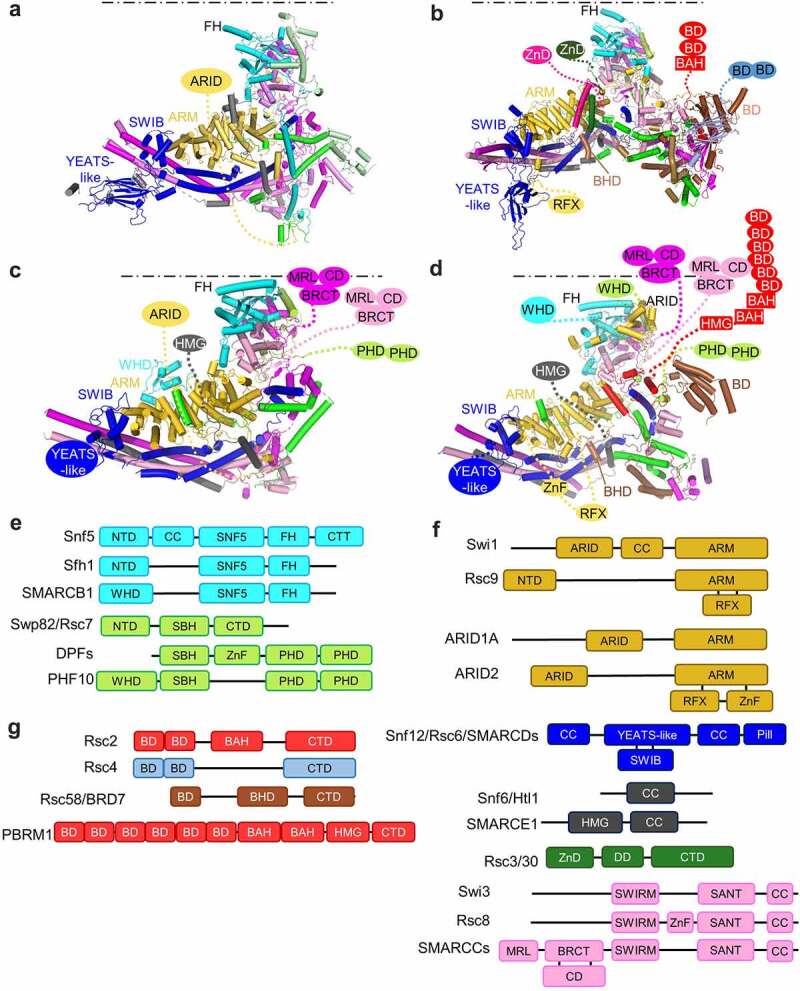
**Structures and the compositions of the SRM of the SWI/SNF family complexes**. (a-d) Structures of the SRM of SWI/SNF (a), RSC (b), BAF (c) and PBAF (d). The positions of the bound nucleosomes are schematically illustrated as dotted lines. (e-g) Domain organization of the subunits at the NBL (e), DBL (f) and HBL (g). DPFs: DPF1, DPF2, and DPF3; SMARCCs: SMARCC1 and SMARCC2; SMARCDs: SMARCD1, SMARCD2 and SMARCD3. SBH, Snf5-binding helix; BHD, BDR7-homology domain. MRL, Mar-like domain.

The NBL of the SRM makes direct contacts to the acidic patch of the nucleosome (Figure 2a-d). The NBL is highly conserved, mainly formed by Snf5 in SWI/SNF, Sfh1 in RSC, SMARCB1 (also named as INI1 and BAF47) in BAF and PBAF (Figure 3a-e). The C-terminal helix of Snf5/Sfh1/SMARCB1, termed as the finger helix (FH), protrudes out of the body of the NBL, and packs against the H2A-H2B surface through multiple arginine residues (Figure 2d). Arg370 in SMARCB1, equivalent to Arg669 of Snf5 and Arg397 in Sfh1, works as the canonical arginine anchor, whereas Arg366, Arg373, Arg374, Arg376, and Arg377 in SMARCB1 also contribute to the binding. Mutations of the finger helix reduce the nucleosome remodeling activity of SWI/SNF and RSC in vitro, and cell fitness in vivo [18,20]. The finger helix of SMARCB1 is frequently mutated in cancers and intellectual disability disorder in human patients [46]. The action in nucleosome recognition supports the functional importance of the finger helix.

Whereas the motor domain binds the DNA at SHL2, the SnAc domain and finger helix recognize the H2A-H2B acidic patches. The nucleosome sandwiched by the SWI/SNF family complexes remains largely intact. This nucleosome-binding mode is different from the previous model of extensive rearrangement of the DNA-histone contact [31], and supports an ATP-dependent mechanism of chromatin remodeling.

The nucleosome-binding mode suggests mechanisms in regulation of the activity by H2A-H2B. The H2A-H2B dimer is subjected to chemical modifications, variant exchange, and loss in the subnucleosome particles [47]. The SMARCA4 activity is sensitive to mutations of the acidic patch [48]. RSC is preferentially localized to and remodels the H2A.Z nucleosomes [49]. Regulation by H2A-H2B is expected to have an important impact on the remodeling activity, and thus chromatin structure and gene transcription. Further study along this line will be interesting.

## Linker DNA recognition

The SWI/SNF family complexes diversify in recognition of the extranucleosomal linker DNA, and the extended histone tails through the auxiliary subunits in the DBL and the HBL (figure 3f-g) [50,51]. The structures provide a basis to understand the distinct chromatin targeting and gene regulation of the complexes.

Linker DNA-binding is mainly achieved through the DBL submodule (figure 3f). Swi1 in SWI/SNF, Rsc9 in RSC, ARID1A in BAF, and ARID2 in PBAF are homologous proteins, and form the structural core of the DBL. They fold similarly into armadillo (ARM) repeat domains (Figure 3a-d), and potentially interact with different client proteins, as the ARM proteins do in usual [52]. Swi1/ARID1A/ARID2, but not Rsc9, contains a DNA-binding ARID domain. The N-terminal and middle fragments of ARID1A and ARID2, respectively, contain a long intrinsic discorded region, the functional importance of which is, however, not clear yet.

Two well-studied subunits for chromatin targeting are Rsc3 and Rsc30 in RSC. Rsc3 and Rsc30 are transcription factors, and responsible for genome-wide targeting of the complex in yeast [15,16]. Rsc3 and Rsc30 form a heterodimer, anchor onto the surface of DBL, and project the N-terminal DNA-binding ZnDs to the linker DNA of the nucleosome [20] (Figures 1b-b). Notably, the overall architecture of RSC orients the motor and the Rsc3/30 subunits, such that the ZnDs are close to the nucleosomal linker DNA at the exit side (Figure 1b). The structure provides the rationale for the recruitment of the RSC by the Rsc3 and Rsc30 to the nucleosome free region (NFR) DNA at the promoter. In doing so, the motor slides the nucleosome away from the Rsc3/30 binding site, potentially opening up the NFR and promoting gene transcription.

Unlike RSC, the BAF and PBAF complexes do not carry a stable TF subunit. We speculate that various TFs bind to the DBL in a manner analogous to Rsc3 and Rsc30 (figure 1f). In support of this idea, the glucocorticoid receptor directly interacts with the ARM domain of ARID1A [53]. In contrast, PBAF selectively mediates ligand-dependent transactivation by VDR (vitamin D3 receptor) [50]. More studies of TF binding to the complexes are of great interest in illustration of the specific functions of the human complexes in response to different cellular signals.

Another salient element of the DBL involved in DNA recognition is the SWIB domain of Snf12 in SWI/SNF, Rsc6 in RSC, and SMARCDs in BAF and PBAF (Figure 3a-d). The SWIB domain is exposed, and positioned close to the exit linker DNA. SWIB is proposed to interact with TFs, such as CEBPε and glucocorticoid receptor [54,55]. Mutation of the SWIB in SMARCD2 fails to recruit CEBPε, and is associated with granulocyte differentiation defect in mice [56]. Snf12/Rsc6/SMARCDs also share a conserved YEATS-like domain (figure 3f), which is adjacent to the SWIB domain, but far away from the histone tails. Genetic experiments indicate a role of this domain in mediating gene regulation [18], which is possibly achieved through binding to TFs.

The combination of varied DNA-binding elements provides the basis for the complexes to recognize DNA (figure 3f). Unique in PBAF, ARID2 has an additional C2H2-type Zinc finger domain (Figure 3d-f), which is important for the function of the protein in cells [57]. Absence in the yeast proteins, human SMARCCs (SMARCC1 and SMARCC2) have a N-terminal module containing the DNA-binding MarR-like domain, a chromodomain and a BRCT domain [58] (Figure 3c, d-f) [58]. Likewise, human SMARCE1 evolves to have an HMG domain, which may bind duplex or four-way junction DNA [59]. Hence, the human BAF complex contains at least seven domains for DNA-recognition (Figure 3c), including the ARID, HMG, WHD, SWIB, YEATS-like, and two MarR-like (MRL) domains. The PBAF complex has 10 domains (Figure 3d), including the ARID, HMG, RFX, ZnF, SWIB, YEATS-like, two MarR-like, and two WHD domains. Compared with BAF, PBAF carries additional RFX and ZnF domains in ARID2, and subtle differences in the ARM and ARID domains. The distinct elements confer the specificity in chromatin targeting and gene regulation.

The structure of the DBL also provides insight into the stability of the complexes. Absence in BAF, the BRD7-homology domain (BHD) of BRD7 in the PBAF complex fills in the gap between the ARM domain of ARID2 and SMARCD1 (Figure 3d). A recent study indicates that loss of SMARCE1, which drives clear cell meningioma [60], selectively destabilizes the BAF complex, with a mild defect in PBAF [61]. One explanation of the PBAF extra stability is that the presence of BRD7 consolidates the DBL.

The structure also shed lights on the assembly of human GBAF complex. BRD9, a specific subunit of the GBAF complex, is highly conserved to BRD7, suggesting a similar role in SMARCD1 binding and DBL stabilization. Consistent with the structural role, BRD9 degradation or deletion, but not the bromodomain inhibitors, impairs growth of the rhabdoid tumor and synovial sarcoma cell lines [8,62,63]. The ARM domain is the structural core of the DBL (Figure 3a-d). However, GBAF lacks the ARM domain and the SMARCE1 subunit [8,9]. It also lacks SMARCB1 to assemble the NBL. These analyses suggest that although BAF and PBAF are assembled analogously, the GBAF complex is probably organized very differently.

## Histone tail recognition

Histone tails and their modifications provide rich sources of chromatin cues to recruit the SWI/SNF family enzymes. In particular, RSC and PBAF contain several subunits involved in histone-tail recognition (Figure 3g). RSC contains six typical histone-binding domains in the SRM (Figure 3b), including two bromodomains and one BAH domain in Rsc2, two bromodomains in Rsc4, and one bromodomain in Rsc58. The human PBAF complex contains 11 typical histone-binding domains in the SRM (Figure 3d), including six bromodomains and two BAH domains in PBRM1, two PHD domains in PHF10, and one bromodomain in BRD7. Rsc2 and PBRM1 are functional homologs, sharing little sequence conservation except the histone-binding domains, whereas BRD7 and Rsc58 show conservation throughout the whole proteins.

Remarkably, the histone-tail binding domains in RSC and PBAF are clustered in space at the HBL submodule, in support of a super histone-tail recognition module for combinatory reading of the histone modifications (Figure 3b-d). The structures suggest that varied histone-binding domains reach out from the HBL through flexible linker sequences, projecting close to the nucleosome and potentially cooperatively sensing the chromatin cues. In addition to the acetylated histones, the bromodomain of BRD7 also binds to acetylated VDR, a non-histone ligand, which affects genome-wide changes of PBAF targeting [64]. Relative to the BAF complex, the presence of HBL in PBAF may confer this complex with the required diversity and sensitivity in response to the histone and non-histone modification signals.

## Structural basis of disease-associated mutations of human SWI/SNF

The BAF and PBAF subunits are frequently mutated in cancers and neurodevelopmental diseases. Depending on the specific cellular context, they can act as tumor suppressors or oncogenes. For instance, the loss of SMARCA4 is found in >90% small cell carcinomas of the ovary, hypercalcemic type (SCCOHT) [65,66]. In contrast, SMARCA4 loss inhibits the growth of acute myeloid leukemia cells [67], melanoma [68,69], and SMARCA2-deficient esophageal squamous cell carcinoma (ESCC) [70]. In-depth understanding of the structure and function of the BAF and PBAF complexes is essential for deciphering the disease mechanisms and the development of therapeutic approaches.

**Figure 4.**
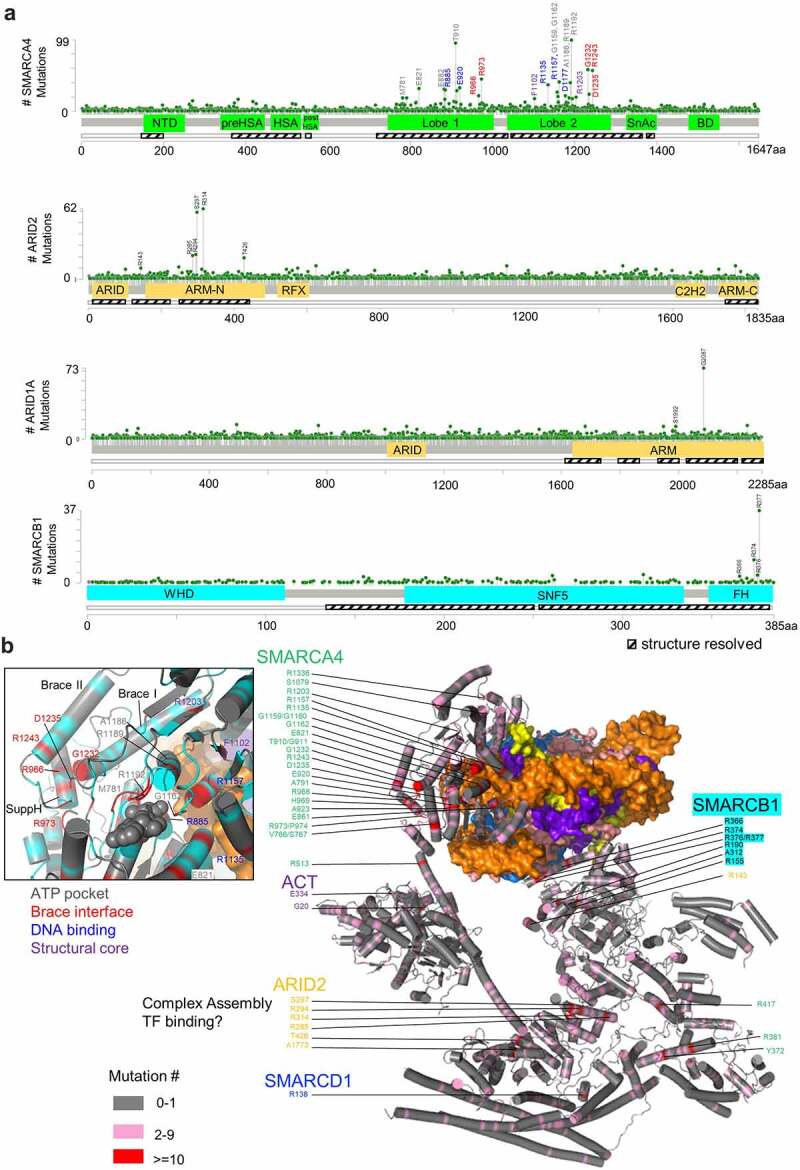
**Mapping of the missense mutations in the human SWI/SNF complexes**. (a) Mapping of the missense mutations of SMARCA4, ARID1A, ARID2 and SMARCB1 into the 1D domain organization diagrams. The data are retrieved from cBioportal and COSMIC. (b) Mapping of the missense mutations of the subunits of PBAF into the 3D structure. The frequently mutated ones (≥10 cases in the cBioportal and COSMIC databases) are labeled. The inset shows the region around the Brace-interface of the motor for further analysis. The hotspot mutations of SMARCA4 motor are classified into 4 groups and color-coded.

SMARCA4, ARID1A, ARID2, and SMARCB1 are recurrently mutated, with over 3700, 3100, 2300 and 700 missense mutations reported in the cBioPortal and COSMIC database, respectively. The mutations occur throughout the whole coding sequences (Figure 4a). The high-resolution structures enable us to map the disease-associated mutations onto the three-dimensional space (Figure 4b). The significance of most of the missense mutations, those occurring at low frequencies in particular, is not clear. The ones with high mutation frequency cluster at three biological important regions, including the C-terminal finger helix of SMARCB1, the motor domain of SMARCA4, and the ARMs of ARID1A and ARID2, which disrupt nucleosome binding, the motor activity, and the structural integrity, respectively.

SMARCB1 is a relatively smaller protein with three major domains (Figure 4a), and its mutation rate per residue is comparable to that of ARID2. Complete inactivation of SMARCB1 destabilizes the BAF and PBAF complexes on chromatin, loses the Polycomb antagonism, and drives malignant rhabdoid tumors (MRTs) [71–73]. More subtle missense mutations, which do not generally lead to loss of the protein or disassembly of the complex, are found in a wide spectrum of cancer types, with >75% of them mappable on the structure. The frequently mutated residues, including Arg366, Arg374, Arg376, and Arg377, cluster at the C-terminal finger helix, which binds to H2A-H2B of the nucleosome, and is important for the chromatin remodeling activity as discussed above.

SMARCA4 is a large, multi-domain protein with 1647 residues (Figure 4a). Above 60% of the missense mutations of SMARCA4 are mappable on the recent structure of PBAF. The mutational hotspots cluster at the motor domain, and are classified into four groups: the ATP-binding pocket, the Brace-helix interface, the DNA binding sites and the structural core. Hotspot mutations, such as Met781 (Motif I), Glu821 (Motif Ia), Thr910 (Motif III), Gly1162 (Motif V), Arg1189 (Motif VI) and Arg1192 (Motif VI), map to the canonical helicase-motifs, which are involved in ATP binding and hydrolysis, as previously studied [74]. Ala1186 locates at the helix carrying Motif VI (Arg1189), and its mutations are expected to perturb the structure of Motif VI to varied levels. Consistent with this notion, the subtle A1186T mutation leads to a partial loss of the activity of the enzyme [75].

Interestingly, five hotspot mutations occur at the novel interface around the Brace-helices outside the canonical helicase-motifs (Figure 4b). Arg966 and Arg973 are mapped around the SuppH, whereas Gly1232, Asp1235, and Arg1243 mapped to the Brace helices and the surrounding loop. The cancer-associated mutations do not disrupt the structural integrity of the complex but led to substantial declines of the remodeling activity in vitro [25]. Different from the A1186T mutation, cancer cells carrying homozygous G1232D or G1232S mutations depend on SMARCA2 for survival [75], supporting the complete functional inactivation of SMARCA4. Patients carrying the brace-helix-interface mutations show low mutational burden [76], suggesting these mutations potentially drive cancer development. It will be very interesting to dissect how these mutations perturb the chromatin structure and the transcription program in vivo, and relate to the disease conditions.

Recurrent mutations are also found at the sites close to the nucleosomal DNA, such as those of Arg885, Glu920, Arg1135, Arg1157, and Asp1177. They are expected to interfere with DNA binding. Phe1102 and Arg1203 are recurrently mutated. They are buried inside the central β-sheet of Lobe2, mutations of which are expected to disrupt the integrity of the enzyme (Figure 4b). Mutations of the SnAc domain are also repeatedly found in cancer patients [76]. It remains to be illustrated how the SnAc domain impacts the disease conditions.

ARID1A and ARID2 are the featured subunits of BAF and PBAF, with 2285 and 1835 amino residues, respectively. ARID1A is subjected to widespread truncating mutations. The nonsense mutation of Y2254* leads to deletion of the last armadillo repeat of the ARM domain and ARID1A disassociation [77], suggesting any preceding truncations result in similar disruption. Moreover, over 3000 missense mutations in ARID1A, and 2000 missense mutations in ARID2 are identified. A few hotspots are identified at the ARM domains (Figure 4a). Ser297, Arg294 and Arg314 of ARID2 map to a helix binding to the preHSA domain of SMARCA4 in PBAF (Figure 4b). Interestingly, Ser1992 in ARID1A, which is frequently mutated, maps a position equivalent to the mutational hotspot of Ser297 in ARID2, suggesting a strategy position for the function of the complexes. We speculate that in addition to complex assembly, ARID1A and ARID2 may be involved in recruitment of transcription factors. Identification of the specific factors interacting with ARID1 and ARID2 is essential for understanding the fine-tuning of transcriptional control through the specific chromatin remodeling complexes.

Likewise, PBRM1 is one of the most commonly mutated genes in clear cell renal cell carcinoma (ccRCC) [78]. It is a peripheral component of PBAF, and incorporated into the HBL through a small piece of ~100 amino residues at the extreme CTD [25]. The structure suggests that any truncating mutations preceding the CTD lead to complete loss of the PBRM1 subunit, and formation of a defective PBAF complex, which may relocate on chromatin and deregulate gene transcription, promoting ccRCC development [79–81].

Mutations of the mSWI/SNF subunits are also found in neurodevelopmental disorders, such as Coffin-Siris syndrome (CSS) and Nicolaides-Baraitser syndrome (NCBRS) [10]. Heterozygous mutations of the finger helix of SMARCB1 perturb neuronal differentiation in a human induced-pluripotent stem cell system [46]. Whereas ARID1A and SMARCA4 have more dominant roles in cancer, their closely paralogs, ARID1B and SMARCA2, frequently mutated in CSS and NCBRS, respectively. This tissue specificity of SMARCA2 and ARID1B may arise because of the increasing expression upon neuronal differentiation [82]. The mutations of SMARCA2 in NCBRS disrupt the ATP-binding pocket, the Brace interface, and the DNA-binding interface, as occurring in cancer [10,25,83]. ARID1B is mutated at the majority of the cases of CSS [84,85], which mostly result in truncated protein and probably disassembly of the complex. The structures of human SWI/SNF complexes pave the way to elucidate the disease mechanism associated with the numerous mutations found in patients.

## Future perspective

The structures of the SWI/SNF family complexes are long-sought after, and a series of breakthroughs have been achieved in recent years [17–26]. These studies reveal that the complexes act commonly through the conserved motors to slide the nucleosome, but are targeted by the distinct auxiliary subunits to achieve specific gene expression. The tripartite modularity of motor-regulation-recruitment is generally appliable to all of the SWI/SNF family complexes.

The targeting subunits are interweaved into the SRM, onto which different histone-binding domains are tethered, and different TFs can dock. The combination of various DNA-binding elements to provide the basis to recognize DNA, and the HBL submodule serves as a super histone-tail recognition module for reading of the histone modifications. In particular, the prototype structure of yeast RSC suggests that the human complexes bind to the exit DNA of the nucleosome through various transcript factors, and thus slide the nucleosome away from the DNA-recognition site. A notable feature of these complexes is the recognition of both acidic patches of the nucleosome, which suggest possible mechanisms in regulation of the activity by H2A-H2B.

The human mSWI/SNF complexes are frequently mutated in cancer. Loss of the peripheral subunits, such as SMARCB1, PBRM1, and SMARCE1, results in formation of defective complexes, which delocalize on chromatin, deregulate gene transcription, and potentially are oncogenic [8,9,61,62,73]. The recent high-resolution structures bound to the nucleosomes mark the beginning to fully understand their functions and molecular mechanisms in cells.

Importantly, targeting mSWI/SNF in TF-dependent cancer is a promising strategy for drug development [86]. However, it is largely unclear how various TFs interact with a particular mSWI/SNF complexes (BAF, PBAF, and GBAF), and with a particular paralog subunit, such as SMARCD1, SMARCD2, and SMARCD3. It also remains elusive how the chromatin cues, such as histone modification and histone variants, recruit and regulate the mSWI/SNF. Many of the subunits of the human complexes contain different splicing isoforms, such as those found in SMARCB1, SMARCE1 and ARID1A [87–89]. The importance of the splicing variants remains largely unexplored. The answers to these questions will elucidate the specific function and mechanism of action of these important enzymes in cells, and facilitate the development of more selective and potent therapeutic agents in the future.
